# Is Booster Dose Strategy Sufficient for Omicron Variant of SARS-CoV-2?

**DOI:** 10.3390/vaccines10030367

**Published:** 2022-02-26

**Authors:** Vivek P. Chavda, Vasso Apostolopoulos

**Affiliations:** 1 Department of Pharmaceutics and Pharmaceutical Technology, L M College of Pharmacy, Ahmedabad 380008, Gujrat, India; vivek.chavda@lmcp.ac.in; 2 Institute for Health and Sport, Victoria University, Melbourne, VIC 3030, Australia; 3 Immunology Program, Australian Institute for Musculoskeletal Science (AIMSS), Melbourne, VIC 3021, Australia

**Keywords:** Omicron variant, SARS-CoV-2, booster dose, vaccine

## Abstract

The Omicron variant of SARS-CoV-2 is emerging in communities where people were previously infected with SARS-CoV-2 and are now being vaccinated, or where many people have received two or three coronavirus vaccination doses. More than 130 countries around the globe have implemented booster dose programs for tackling omicron endemics. Despite early findings shows that booster doses may improve omicron protection, more research is needed to establish vaccination efficacy. This short communication tries to critically discuss the research work findings around booster dose strategy for omicron endemics.

“A booster dose is an injection after by which a vaccine has been administered and induced immunity. Booster dose is to boost the immune response sometime later after primary immunization. In the case of coronavirus disease 2019 (COVID-19), it was recommended to have 2 injections of most of the vaccines, and 6 months later they are seeing a decline in immunity [1], hence they are recommending a booster dose. The objective of a booster dose is to restore vaccine effectiveness from the immune protection point that deemed no longer sufficient.” A booster immunization offers re-exposure to the immunizing antigen after initial immunization. It is intended to improve protective immunity to the pathogen following a decline in immunity over time (Figure 1) [2,3]. As per the world health organization (WHO), “Additional doses of a vaccine may be needed as part of an extended primary series for target populations where the immune response rate following the standard primary series is deemed insufficient. The objective of an additional dose in the primary series is to enhance the immune response to establish a sufficient level of effectiveness against disease. In particular, immunocompromized individuals often fail to mount a protective immune response after a standard primary series, but also older adults may respond poorly to a standard primary series with some vaccines [4,5,6].” Following the primary vaccination schedule, the need for a booster dose is assessed in several ways, such as via concentration of neutralizing antibodies and T cell immunity as well as, memory B and T cell responses [7,8]. In some cases, when the patient has high levels of neutralizing antibodies and receives a booster dose, they may face an inflammatory Type III hypersensitivity reaction known as an *“Arthus reaction*” that demands a suitable time gap between immunization and the booster dose for its avoidance [9].

The WHO named the newly categorized variant of concern (VOC), the Omicron variant, a “severe acute respiratory syndrome coronavirus 2 (SARS-CoV-2)”, due to its ominous earlier prestige [10]. First detected in South Africa on December 1st 2021, it is now present in almost 89 countries around the world. The assertion that Omicron has proceeded to distribute in places with a high incidence of prior SARS-CoV-2 infestation and in well-vaccinated regions is notably alarming, and top experts conclude that Omicron may outpace Delta and become the causative agent of SARS-CoV-2 globally [11]. Meng and colleagues reported, “Omicron appears to have gained significant evasion from neutralizing antibodies whilst maintaining sensitivity to antiviral drugs targeting the polymerase. Omicron has shifted cellular tropism away from transmembrane protease, serine 2 expressing cells that are enriched in cells found in the lower respiratory and gastrointestinal tracts, with implications for altered pathogenesis [12].” In addition, with over 50 mutations in its genetic code, of which 30 are in the gene encoding the Spike protein, the SARS-CoV-2 surface protein facilitates its interaction with angiotensin-converting enzyme 2 (ACE2) receptors to facilitate infection and entry into host cells. As such, the Spike protein is used in most currently approved vaccines, however, significant concerns about Omicron’s enhanced transmissibility and immune escape have been raised [13]. In the research facility, the Omicron pseudovirus replicated 70 times faster than Delta and the original SARS-CoV-2 strain, according to a study by the University of Hong Kong [14]. Despite this, it propagated roughly ten times less rapidly in lung tissue than the original strain, which might indicate a milder illness [15]. The Omicron variant is naturally resistant to neutralization by plasma from both convalescent patients and those who have been immunized with one of the four most commonly rolled-out COVID-19 vaccines (Pfizer, AstraZeneca, Moderna, and Johnson & Johnson) [16]. The efficacy of the vaccine is 34-fold less for immune protection with two doses of the vaccine. Even the serum from those who have been immunized (33 to 40% immune protection) and boosted with mRNA-based vaccines (80 to 85% immune protection) significantly reduced neutralizing antibody efficacy against the Omicron variant [17]. According to Agrawal and Colleagues, “Omicron represents a significant challenge to the existing two-dose vaccination strategy presently adopted by many countries globally (17 to 22-fold reduction in neutralization titers)”, as such a third booster vaccine is recommended [14]. Whilst the other VOCs Beta and Gamma also represented challenges to vaccine efficacy, two defining features of Omicron provide additional concerns. Firstly, as observed herein, the fold evasion to humoral immunity is significantly greater with Omicron than all other VOCs. Secondly, unlike Beta and Gamma, Omicron is gaining momentum in global prevalence in areas where Delta dominated in late 2021. Whilst boosters utilizing the same Clade A Spike may increase antibody titers to Omicron, the development of variant-specific boosters may be more pragmatic in the longer term if Omicron persists. The latter will be very important in those groups that may have a limited titer, such as in the elderly or immunocompromised. Fortunately, for the latter at-risk groups, certain immunotherapeutic treatments including Sotrovimab appear to maintain potency and remain relevant for treatment in Omicron cases. A study conducted by Lewnard and colleagues in the USA demonstrated that SARS-CoV-2 infection with suspected Omicron variant transmission were linked with a significantly lower incidence of severe clinical outcomes and shorter hospital stays during a time of mixed Delta and Omicron variant flow [18]. Sera collected three months following a second BNT162b2 immunization had 27-fold lower neutralizing antibody titers against Omicron than D614G mutant sera [19]. The neutralization titer in convalescent sera from Alpha and Delta patients is likewise lowered. Some Delta patients, on the other hand, show relatively intact antibody-neutralizing activity up to the level of a 3-month double BNT162b2 vaccination. The ACE-2 decoy is another method for neutralizing the virus that is not reliant on mutational escape, and Omicron is likewise susceptible to the modified ACE2 [20]. “T-cells respond to the whole of the spike protein, so they are less likely to be affected by a few mutations, and how omicron will interact with populations with low immunity against COVID-19 remains to be seen”, as pointed out by Dunachie and Burki [21]. As a booster dosage, many COVID vaccinations have been demonstrated to be safe and efficacious. The Cov-Boost experiment investigated the use of seven different vaccines as boosters following two doses of either the AstraZeneca or Pfizer vaccines, including AstraZeneca, Curevac, Johnson & Johnson (Janssen), Moderna, Novavax, Pfizer, and Valneva. The trial demonstrated that all vaccinations (except Curevac, which was discontinued) increased the immunological response; however, the number of antibodies varied greatly depending on the vaccine combination [22,23,24]. According to a study conducted by Sheikh and colleagues, “Omicron is associated with a two-thirds reduction in the risk of COVID-19 hospitalization when compared to Delta. Whilst offering the greatest protection against Delta, the third/booster dose of vaccination offers substantial additional protection against the risk of symptomatic COVID-19 for Omicron when compared to ≥25 weeks post second vaccine dose [25,26].” Serum specimens from 88 individuals who requested the Moderna COVID-19 vaccine, 111 people who obtained the Pfizer COVID-19 vaccine, and 40 recipients of the Johnson & Johnson COVID-19 vaccine showed almost negligible neutralizing activity towards Omicron. Serum samples from people who had been boosted with mRNA vaccinations, on the other hand, showed recovered neutralization activity that was only 4-6 times weaker against Omicron than against the wild-type pseudovirus [27]. Zhang and colleagues performed pseudovirus assays along with vaccinated sera that were suppressed tenfold more effectively by triply vaccinated sera than by doubly vaccinated sera [28]. As a result, while the omicron spike enhances immune evasion, booster vaccines can help guard against omicron spike-mediated viral entrance. Similarly, Pajon and colleagues achieved a 20 times higher neutralization antibody titer with an mRNA booster dose than second dose of the vaccine [29]. According to a recent research study, “A 50 μg boost increased Omicron neutralization titers and may substantially reduce the risk of symptomatic vaccine breakthrough infections [30].” Recent research suggests that “Omicron is antigenically distant from the original SARS-CoV2 vaccine strain than the previously most distant strains beta and delta that demands a vaccine booster to get substantial antibody neutralization titers [10,31,32].” With three doses of Valneva’s inactivated adjuvanted COVID-19 vaccine, the candidates demonstrated 87 % protection against the omicron variant of SARS-CoV-2. The vaccine candidates also demonstrated a broad T cell response in the Phase 3 trial [33].

The supplemental dose of mRNA vaccines now distributed may result in more rigorous and long-lasting host defense, precluding the need for additional boosters. Furthermore, the development of novel vaccines that provide protection against less mutable coronavirus proteins may result in broader protection [34]. Additionally, SARS-CoV-2 transformation and epidemiological studies have been uncertain, and being able to respond, such as with vaccination programs and booster doses of vaccination programs are needed, as well as non-vaccine mitigation strategies, remains essential in helping to reduce COVID-19-associated hospitalizations and deaths [35]. The Omicron VOC poses a major danger to several currently developed COVID-19 vaccines and therapeutics, necessitating the establishment of innovative therapies that anticipated SARS-evolutionary CoV-2. Booster jabs appear to improve defense, but their longevity, effectiveness, and potential to thwart the new type are unknown. Complete genomic and pharmacological understanding of Omicron VOC is needed, which defines clinical phenomes and therapeutics, tracks the dynamics of genetic changes, and transfers COVID-19 information into new variants. The implications of clinical and translational medicine would be momentous in tackling such issues by providing new insights into understanding and forecasting novel variant-associated transmissibility, disease severity, immunological escape, diagnostic, or therapeutic failure.

More than 130 countries around the globe have implemented booster dose programs, although the coverage rates for complete primary vaccination are below 30% in the majority of these countries. The major goal to halt this pandemic is to achieve 100% primary vaccination coverage globally with a secondary objective of full vaccination of elderly and immunocompromised individuals for a substantial reduction in severe disease and mortality. Given the ongoing logistic challenges in global vaccine availability and equity, each nation’s vaccine-booster dosage-policy decisions must balance public health advantages for their populations with support for global equality in vaccine access, which is required to address virus mutation and the effect of the pandemic. Indications of decreasing vaccine efficacy, namely a loss in coverage against severe illness in high-risk groups, necessitates the development of vaccination techniques tailored for severe disease prevention, including the tailored use of booster immunization.

## Figures and Tables

**Figure 1:**
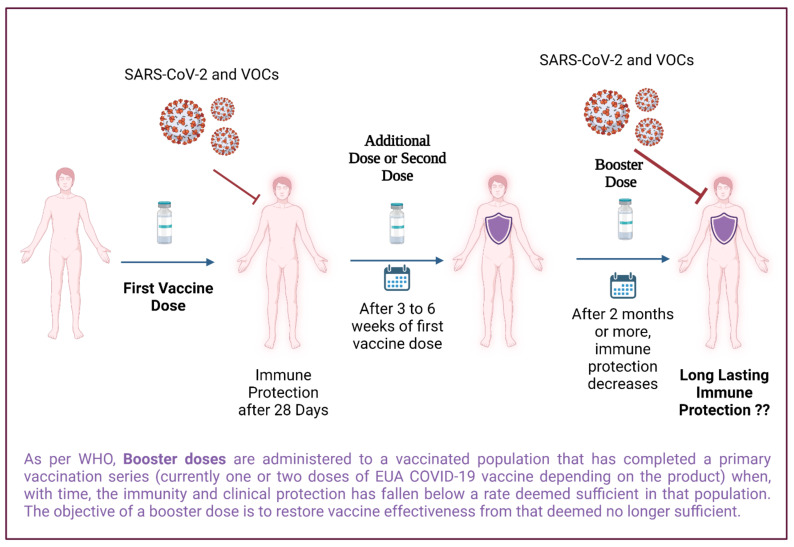
The booster dose strategy against omicron and other VOCs of SARS-CoV-2.

## Data Availability

Not applicable.
